# Types of Glaucoma and Associated Comorbidities Among Patients at King Abdulaziz Medical City, Jeddah

**DOI:** 10.7759/cureus.15574

**Published:** 2021-06-10

**Authors:** Karim Talaat, Obada T Fathi, Saeed M Alamoudi, Muhanad G Alzahrani, Rayan M Mukhtar, Muhammad A Khan

**Affiliations:** 1 Vitreoretinal Surgery - Department of Ophthalmology, King Abdulaziz Medical City, Ministry of National Guard Health Affairs / King Saud bin Abdulaziz University for Health Sciences, Jeddah, SAU; 2 College of Medicine, King Saud bin Abdulaziz University for Health Sciences, Jeddah, SAU; 3 Medical Education, College of Medicine, King Saud bin Abdulaziz University for Health Sciences, Jeddah, SAU; 4 Research, King Abdullah International Medical Research Center, Jeddah, SAU; 5 College of Medicine, King Abdulaziz Medical City, Jeddah, SAU

**Keywords:** hospital epidemiology, prevalence study, primary open angle glaucoma, open angle glaucoma, neovascular glaucoma, medical comorbidities

## Abstract

Aim

To identify the types of glaucoma and associated comorbidities among patients attending the ophthalmology clinic at King Abdulaziz Medical City (KAMC) in Jeddah.

Methods

A cross-sectional study that included all glaucoma patients at KAMC in Jeddah between June 1st, 2016 and November 30th, 2020. Data were collected through retrospective chart review from the electronic medical record system (BestCare) and utilized a structured data collection sheet.

Results

A total of 283 patients met the inclusion criteria. The most common type was primary open-angle glaucoma (POAG; 53%) followed by secondary glaucomas (SG; 26.5%) then childhood glaucoma and primary angle-closure glaucoma (CG, PACG; 7.4%). The majority of secondary glaucoma cases were due to neovascular glaucoma (NVG; 44.9%), followed by phacomorphic glaucoma (17.9%) and phacolytic glaucoma (10.3%). Hypertension (60.8%) and diabetes (58.3%) were the most prevalent systematic comorbidities, and cataract (49.1%) was the most prevalent ocular comorbidity.

Conclusion

POAG was the most common glaucoma type, followed by SG, CG, and PACG. Among secondary glaucoma types, neovascular glaucoma was found to be the most common subtype. Hypertension was the most prevalent comorbid condition.

## Introduction

Blindness is one of the major public health concerns, and as the size of the population increases, this issue will exert a huge burden on public health [1]. Glaucoma is a group of eye diseases that can result in irreversible visual field loss and degenerative optic neuropathy. Usually, it is asymptomatic at early stages, but permanent visual impairment can ensue at late stages [2]. Thus, early checkups and diagnosis followed by the appropriate treatment are crucial [3,4]. In addition, several risk factors are associated with glaucoma, including age, hypertension, and refractive errors [5].

Glaucoma is regarded as the leading cause of irreversible blindness and the second leading cause of blindness worldwide by the World Health Organization (WHO) [6,7]. It is estimated that the pooled worldwide prevalence of glaucoma between the ages of 40-80 is 3.54% [6]. Moreover, the global prevalence of primary open-angle glaucoma (POAG) and primary angle-closure glaucoma (PACG) is 3.05% and 0.50%, respectively. POAG was highest among patients of African ancestry, while those of Asian ancestry had the highest prevalence of PACG [6]. Projections for the worldwide burden of glaucoma have estimated that glaucoma prevalence will rise by 74% from 2013 to 2040, which accounts for 111.8 million patients, most of whom are in Asia and Africa [6]. However, there is insufficient evidence regarding the prevalence of glaucoma and its causes in the Saudi population [7]. The latest estimate on the prevalence of glaucoma in Saudi Arabia is 5.6%, based on a 2019 study in Riyadh governorate which involved 940 subjects across six primary health care facilities [4].

Visual impairment continues to be a major public health concern with a substantial impact on all aspects of quality of life, including physical functioning, emotional distress, and socioeconomic loss [8]. The paucity of data on types of glaucoma and their risk factors in the Saudi population highlights the importance of such studies, which allow for the planning and implementation of blindness prevention programs according to the needs of different populations. This study aims to identify the common causes of glaucoma and associated comorbidities among patients attending the ophthalmology clinic at King Abdulaziz Medical City (KAMC) in Jeddah.

## Materials and methods

This cross-sectional study was conducted at KAMC in Jeddah, Saudi Arabia. It included patients diagnosed with glaucoma attending general ophthalmology clinics between June 1st, 2016 and November 30th, 2020, using a consecutive sampling technique. Patients with other types of optic neuropathy were excluded from the study. A retrospective chart review was conducted using electronic medical records and a structured data collection sheet. The data collection sheet consisted of socio-demographic variables like age, gender, and BMI. Moreover, clinical variables such as glaucoma type and etiology, visual acuity, treatments, and comorbidities, were recorded. Using the International Council of Ophthalmology classification, the best corrected visual acuity (BCVA) was categorized into normal vision as 6/6 - 6/7.5, near-normal vision as 6/9.5 - 6/18, moderate vision loss as 6/21 - 6/48, severe vision loss as 6/60 - counting fingers (CF), near-blindness is hand motion (HM) - light perception (LP), and blindness as no light perception (NLP) [9]. For children from 2 months to 24 months, visual acuity was assessed by fixating eyes on an object and following it. The visual acuity of infants aged less than one month was assessed by blinking response to light [9]. Variables were represented as median and interquartile ranges. The data were compiled in Microsoft Excel 2016 and analyzed using IBM Statistical Package for the social sciences (SPSS) version 25.0 (IBM Corp., Armonk, NY, USA). Qualitative variables were presented using descriptive statistics in the form of categories and summarized as frequencies and percentages. Data comparison was interpreted using the Chi-square test and Fisher exact test, and a p-value that is less than 0.05 was considered significant.

All patients’ data were kept confidential, and ethical approval was obtained from the Institutional Review Board at King Abdullah International Medical Research Centre (KAIMRC), National Guard Health Affairs (NGHA), Jeddah, Saudi Arabia (Reference number: JED-21-427780-3866, January 26th, 2021).

## Results

A total of 283 patients fulfilled the inclusion criteria, 133 (47%) of which were males, and 150 (53%) were females. Our sample noted that 59.9% of the patients had bilateral disease, while 40% had unilateral disease (p < 0.001). The median (IQR) for age was 66 years (57-74), ranging from two months to 101 years old, BMI 29 kg/m^2^ (25-33), and intraocular pressure of 16 mmHg (13-20) in both eyes (Table 1).

**Table 1:** Proportions of study sample by age, BMI, and IOP.

		Median	IQR
Age (months)		66	57-74
BMI (Body Mass Index)		29	25-33
Right eye IOP (Intra-Ocular Pressure)		16	13-20
Left eye IOP (Intra-Ocular Pressure)		16	13-20

**Figure 1:**
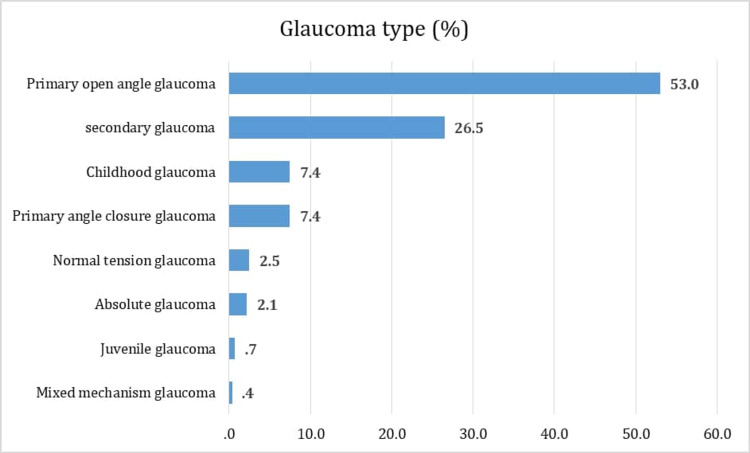
Distribution of glaucoma types

The most common glaucoma types were primary open-angle glaucoma (POAG; 53%) followed by secondary glaucoma (SG; 26.5%), then childhood glaucoma and primary angle-closure glaucoma (CG, PACG; 7.4%). Normal-tension glaucoma (NTG) accounted for 2.5%, absolute (unspecified) glaucoma accounted for 2.1%, and both juvenile and mixed mechanism glaucoma represented 0.7% and 0.4% of the sample, respectively (Figure 1). Additional data, including affected eye analysis, are shown in Table 2.

**Table 2:** Distribution of gender and affected eyes between glaucoma types Oculus Dexter (OD) for the right eye, Oculus sinister (OS) for the left eye, Oculus Uterque (OU) for both eyes.

		Glaucoma Type																	
		Primary open angle glaucoma		Secondary glaucoma		Primary angle closure glaucoma		Normal tension glaucoma		Childhood glaucoma		Mixed mechanism glaucoma		Juvenile glaucoma		Absolute glaucoma			p-value
		n	%	n	%	n	%	n	%	n	%	n	%	n	%	n	%		
Eye affected																		<0.001	
	OD	13	22.0%	29	49.2%	8	13.6%	0	0.0%	4	6.8%	1	1.7%	0	0.0%	4	6.8%		
	OS	16	29.6%	26	48.1%	7	13.0%	0	0.0%	3	5.6%	0	0.0%	0	0.0%	2	3.7%		
	OU	121	71.6%	20	11.8%	6	3.6%	7	4.1%	13	7.7%	0	0.0%	2	1.2%	0	0.0%		
Gender																		0.087	
	Female	77	51.3%	34	22.7%	16	10.7%	3	2.0%	15	10.0%	0	0.0%	1	.7%	4	2.7%		
	Male	73	54.9%	41	30.8%	5	3.8%	4	3.0%	6	4.5%	1	.8%	1	.8%	2	1.5%		

**Figure 2:**
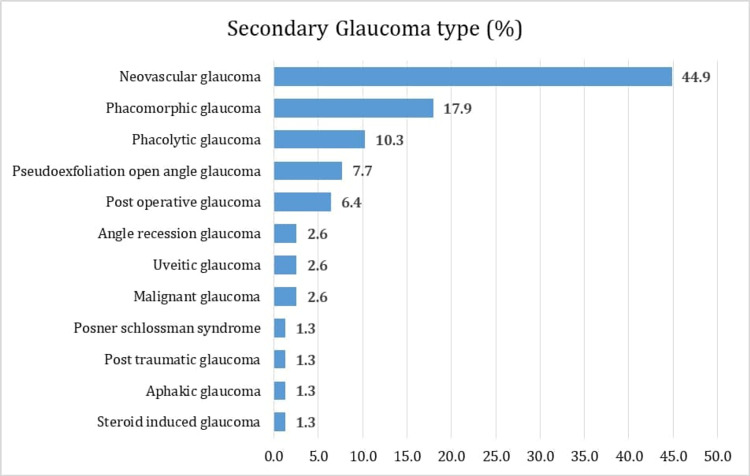
Distribution of secondary glaucoma types

Further analysis of secondary glaucoma patients into subcategories revealed that most cases were due to neovascular glaucoma (NVG, 44.9%). Other types of secondary glaucoma included phacomorphic glaucoma (17.9%) and phacolytic glaucoma (10.3%). Additional types of glaucoma that were seen less frequently included angle recession glaucoma (2.6%), uveitis glaucoma (2.6%), malignant glaucoma (2.6%), post-traumatic glaucoma (1.3%), and aphakic glaucoma (1.3%) (Figure 2). Additional data about secondary glaucoma types are shown in Table 3.

**Table 3:** Distribution of gender and affected eyes among patients suffering from secondary glaucoma Oculus Dexter (OD) for the right eye, Oculus sinister (OS) for the left eye, Oculus Uterque (OU) for both eyes.

		Secondary Glaucoma																																	
		Neovascular glaucoma		Pseudoexfoliation open-angle glaucoma		Malignant glaucoma		Phacolytic glaucoma		Phacomorphic glaucoma		Steroid-induced glaucoma		Uveitic glaucoma			Angle recession glaucoma			Aphakic glaucoma			Post-traumatic glaucoma			Posner Schlossman syndrome			Postoperative glaucoma			p-value		
		n	%	n	%	n	%	n	%	n	%	n	%	n	%	n		%	n		%	n		%	n		%	n		%			
Eye affected																															0.491		
	OD	14	48.3%	1	3.4%	1	3.4%	2	6.9%	7	24.1%	1	3.4%	1	3.4%	0		0.0%	0		0.0%	0		0.0%	1		3.4%	1		3.4%			
	OS	13	48.1%	1	3.7%	1	3.7%	2	7.4%	5	18.5%	0	0.0%	0	0.0%	2		7.4%	0		0.0%	1		3.7%	0		0.0%	2		7.4%			
	OU	8	36.4%	4	18.2%	0	0.0%	4	18.2%	2	9.1%	0	0.0%	1	4.5%	0		0.0%	1		4.5%	0		0.0%	0		0.0%	2		9.1%			
Gender																															0.725		
	Female	14	38.9%	3	8.3%	1	2.8%	5	13.9%	9	25.0%	0	0.0%	1	2.8%	0		0.0%	0		0.0%	0		0.0%	0		0.0%	3		8.3%			
	Male	21	50.0%	3	7.1%	1	2.4%	3	7.1%	5	11.9%	1	2.4%	1	2.4%	2		4.8%	1		2.4%	1		2.4%	1		2.4%	2		4.8%			

**Figure 3:**
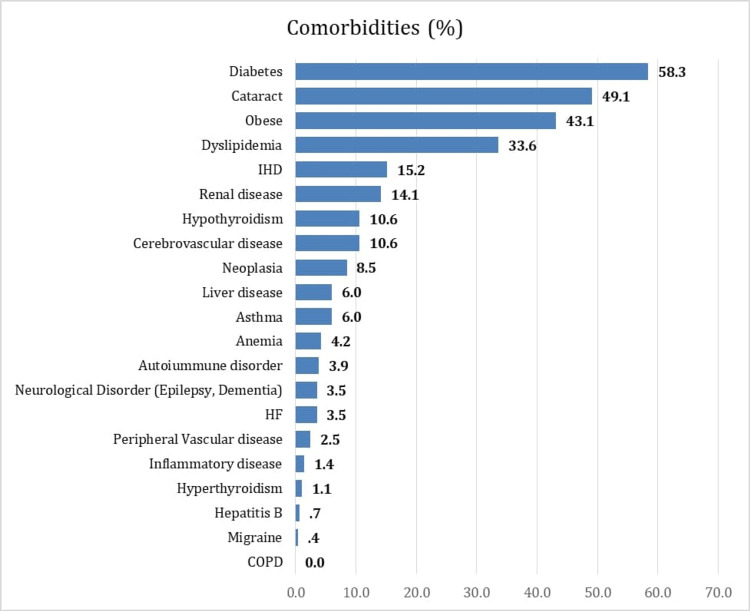
Prevalence of comorbidities among glaucoma patients Ischemic Heart Disease (IHD), Heart Failure (HF), Chronic obstructive pulmonary disease (COPD).

The most common associated systematic comorbidities among patients diagnosed with glaucoma were hypertension (60.8%), diabetes (58.3%), obesity (43.1%), and dyslipidemia (33.6%). Cataract (49.1%) was the most prevalent associated ocular comorbidity. Other comorbidities were also noted in glaucoma patients but were less frequent, such as ischemic heart disease (15.2%), renal diseases (14.1%), hypothyroidism, and cerebrovascular disease (10.6%). Further details are shown in Figure 3.

Analysis of visual acuity showed that most patients had a moderate loss of visual acuity (26.87%), followed by near-normal visual acuity (19.22%), and followed by severe loss of visual acuity (18.15%). Most of the children were able to fix and follow objects with their eyes (0.8%) and blink to light (0.7%), while only a few of them couldn't fix and follow objects (0.5%). Details regarding visual acuity impairment are found in Table 4.

**Table 4:** Visual acuity among glaucoma patients Visual acuity (VA), Right eye (Rt), Left eye (Lt)

	Rt VA		Lt VA		Total VA	
	n	%	n	%	n	%
Normal	45	16.0	45	16.0	90	16.01
Near-normal	53	18.9	55	19.6	108	19.22
Moderate	75	26.7	76	27.0	151	26.87
Severe	50	17.8	52	18.5	102	18.15
Near blindness	19	6.8	20	7.1	39	6.94
Legal blindness	39	13.9	33	11.7	72	12.81
Total	281	100	281	100	562	100

Control of intraocular pressure was achieved by medical, laser, or surgical methods. In terms of medical therapy used in our sample, beta-blockers were the most commonly used agents (72%), followed by carbonic anhydrase (CA) inhibitors (71.3%). Prostaglandins and ⍺2-agonists were used by 61.4% and 60.7% of patients, respectively. Moreover, laser peripheral iridotomy was utilized in 7.4%, while cyclocryocoagulation was used in 4.9%. Regarding surgical procedures, trabeculectomy was performed in 50.1% of patients. Further information is represented in Table 5.

**Table 5:** Distribution of treatment used for every glaucoma type Extracapsular cataract extraction (ECCE)

		Glaucoma Type								
		Primary open angle glaucoma	Secondary glaucoma	Primary angle closure glaucoma	Normal tension glaucoma	Childhood glaucoma	Mixed mechanism glaucoma	Juvenile glaucoma	Absolute glaucoma	p-value
		n(%)	n(%)	n(%)	n(%)	n(%)	n(%)	n(%)	n(%)	
Prostaglandins	No	49(45)	37(33.9)	4(3.7)	3(2.8)	12(11)	0(0)	1(0.9)	3(2.8)	0.062
	Yes	101(58)	38(21.8)	17(9.8)	4(2.3)	9(5.2)	1(0.6)	1(0.6)	3(1.7)	
B-Blockers	No	39(49.4)	25(31.6)	4(5.1)	3(3.8)	5(6.3)	0(0)	2(2.5)	1(1.3)	0.248
	Yes	111(54.4)	50(24.5)	17(8.3)	4(2)	16(7.8)	1(0.5)	0(0)	5(2.5)	
a2-agonists	No	47(42.3)	34(30.6)	9(8.1)	2(1.8)	11(9.9)	1(0.9)	2(1.8)	5(4.5)	0.020
	Yes	103(59.9)	41(23.8)	12(7)	5(2.9)	10(5.8)	0(0)	0(0)	1(0.6)	
Miotic agents (cholinergic)	No	150(53.4)	75(26.7)	19(6.8)	7(2.5)	21(7.5)	1(0.4)	2(0.7)	6(2.1)	0.001
	Yes	0(0)	0(0)	2(100)	0(0)	0(0)	0(0)	0(0)	0(0)	
CA inhibitors	No	41(50.6)	25(30.9)	3(3.7)	3(3.7)	6(7.4)	0(0)	2(2.5)	1(1.2)	0.215
	Yes	109(54)	50(24.8)	18(8.9)	4(2)	15(7.4)	1(0.5)	0(0)	5(2.5)	
TNF inhibitor	No	67(36.6)	68(37.2)	12(6.6)	6(3.3)	21(11.5)	1(0.5)	2(1.1)	6(3.3)	0.067
	Yes	0(0)	0(0)	1(100)	0(0)	0(0)	0(0)	0(0)	0(0)	
Mannitol	No	150(53.2)	75(26.6)	20(7.1)	7(2.5)	21(7.4)	1(0.4)	2(0.7)	6(2.1)	0.085
	Yes	0(0)	0(0)	1(100)	0(0)	0(0)	0(0)	0(0)	0(0)	
Trabeculoplasty	No	148(52.9)	75(26.8)	21(7.5)	7(2.5)	20(7.1)	1(0.4)	2(0.7)	6(2.1)	0.774
	Yes	2(66.7)	0(0)	0(0)	0(0)	1(33.3)	0(0)	0(0)	0(0)	
Cyclophotocoagulation	No	149(53)	75(26.7)	20(7.1)	7(2.5)	21(7.5)	1(0.4)	2(0.7)	6(2.1)	0.573
	Yes	1(50)	0(0)	1(50)	0(0)	0(0)	0(0)	0(0)	0(0)	
Cyclocryocoagulation	No	149(55.4)	67(24.9)	20(7.4)	7(2.6)	20(7.4)	1(0.4)	2(0.7)	3(1.1)	<0.001
	Yes	1(7.1)	8(57.1)	1(7.1)	0(0)	1(7.1)	0(0)	0(0)	3(21.4)	
Pan-retinal photocoagulation	No	150(53.4)	73(26)	21(7.5)	7(2.5)	21(7.5)	1(0.4)	2(0.7)	6(2.1)	0.589
	Yes	0(0)	2(100)	0(0)	0(0)	0(0)	0(0)	0(0)	0(0)	
Laser peripheral iridotomy (YAG)	No	148(56.6)	71(27.1)	7(2.7)	7(2.7)	20(7.6)	1(0.4)	2(0.8)	6(2.3)	<0.001
	Yes	2(9.5)	4(19)	14(66.7)	0(0)	1(4.8)	0(0)	0(0)	0(0)	
Minimally invasive procedure	No	150(53.2)	74	21	7	20	1	2	6	0.464
	Yes	0	1	0	0	1	0	0	0	
Sclerectomy - Viscocanalostomy	No	150	75(26.6)	21(7.4)	7(2.5)	20(7.1)	1(0.4)	2(0.7)	6(2.1)	0.085
	Yes	0(0)	0(0)	0(0)	0(0)	1(100)	0(0)	0(0)	0(0)	
Trabeculectomy	No	124(51.7)	70(29.2)	20(8.3)	7(2.9)	11(4.6)	1(0.4)	1(0.4)	6(2.5)	<0.001
	Yes	26(60.5)	5(11.6)	1(2.3)	0(0)	10(23.3)	0(0)	1(2.3)	0(0)	
Vitrectomy	No	150(54.2)	69(24.9)	21(7.6)	7(2.5)	21(7.6)	1(0.4)	2(0.7)	6(2.2)	0.017
	Yes	0(0)	6(100)	0(0)	0(0)	0(0)	0(0)	0(0)	0(0)	
Iridectomy	No	150(53)	75(26.5)	21(7.4)	7(2.5)	21(7.4)	1(0.4)	2(0.7)	6(2.1)	Cannot be calculated
	Yes	0(0)	0(0)	0(0)	0(0)	0(0)	0(0)	0(0)	0(0)	
Retinectomy	No	150(53.2)	74(26.2)	21(7.4)	7(2.5)	21(7.4)	1(0.4)	2(0.7)	6(2.1)	0.904
	Yes	0(0)	1(100)	0(0)	0(0)	0(0)	0(0)	0(0)	0(0)	
Eye evisceration/ eye removal	No	150(53.4)	74(26.3)	21(7.5)	7(2.5)	20(7.1)	1(0.4)	2(0.7)	6(2.1)	0.464
	Yes	0(0)	1(50)	0(0)	0(0)	1(50)	0(0)	0(0)	0(0)	
Phacoemulsification / ECCE	No	148(56.5)	57(21.8)	21(8)	7(2.7)	21(8)	1(0.4)	2(0.8)	5(1.9)	<0.001
	Yes	2(9.5)	18(85.7)	0(0)	0(0)	0(0)	0(0)	0(0)	1(4.8)	

## Discussion

The present study demonstrates the glaucoma profile in patients who presented to KAMC in Jeddah. In addition, we analyzed the pattern of glaucoma among the patients to estimate the prevalence of different types of glaucoma, comorbidities, and clinical characteristics using internationally recognized glaucoma definitions.

No statistically significant difference was noted between males and females in our study as 53% of the sample were female compared to 47% males (P-value = 0.087). This contrasts with a local study which showed a significant difference between genders with a female predominance as 59% of their sample were females (p-value < 0.0001) [10]. Furthermore, we found that most patients had bilateral disease compared to either right or left eye involvement reaching statistical significance (p-value < 0.001). This finding is in line with other studies which showed a significantly greater proportion of bilateral involvement in patients with glaucoma. Moreover, a study done in Saudi Arabia supports our finding where it showed a difference between the two genders - females were 729 (59%), and males were 507 (41%) reaching clinical significance (p < .0001) with 816 (66%) suffering bilateral disease and 420 (34%) suffering unilateral disease (p < 0.0001) [10].

Types

Our study showed that POAG is the predominant subtype, followed by secondary glaucoma types, childhood glaucoma, and PACG. The results are consistent with studies that have been conducted in western countries which reported POAG to be more prevalent (89.0%) than PACG [2]. However, other studies from eastern countries with a high population size reported PACG is more prevalent than primary POAG [11]. Moreover, the Vellore Eye Study (VES) was one of the first studies emphasizing the high burden of angle closure in the Asian population with a prevalence of 10.3% of either occludable angles or angle-closure [12]. Furthermore, a study conducted in Oman reported the prevalence of POAG as 63.5% and PACG as 12% [13]. The low prevalence of closed-angle or narrow-angle glaucoma in their study might be because non-glaucoma specialists tend not to use the gonioscopy routinely to measure the angle. Secondary glaucoma represented 26.5% of the sample. These subtypes result from other pathological, surgical, or traumatic eye lesions. The prevalence of diabetes mellitus in Saudi Arabia contributes to the high incidence of proliferative diabetic retinopathy, which results in the increased prevalence of neovascular glaucoma (44.9%) among the other secondary glaucoma types.

Comorbidities

Multiple studies have shown hypertension to be among the most common comorbid conditions with glaucoma, consistent with our findings [14,15]. However, approximately 61% of our sample had reported hypertension; other local and global studies have shown a much lower prevalence of hypertension at 35% and 37%, respectively [14,15]. The significance of this association is controversial as the literature provides conflicting reports attributed to differences in the ethnic background of the samples being studied since populations of Asian, African, and Caucasian descent have a different prevalence of primary open-angle glaucoma and normal-tension glaucoma [14,16]. Two mechanisms are postulated to explain the relationship between hypertension and open-angle glaucoma. The first is that increased arterial pressure will cause hardening and atherosclerotic changes in the retinal vasculature with subsequent arteriolar narrowing and elevated resistance, which eventually compromises the adequacy of perfusion to the optic disc [14,17,18]. The other mechanism is related to the use of medications to lower blood pressure that might trigger episodes of systemic hypotension, which results in transient reductions in ocular blood supply [14,19,20].

Another common comorbidity in our sample was diabetes mellitus, as seen in 58% of patients, which is higher than local studies in Riyadh and Qassim, which reported a prevalence of 14.9% and 32.8%, respectively [4,15]. The mechanism by which diabetes is linked to glaucoma is believed to arise from impaired autoregulation of retinal and elevated predilection of retinal ganglion cells to programmed cell death [14,21]. The significance of the association between diabetes and glaucoma has been supported by some studies, whereas other articles have failed to demonstrate any significant association [14,22].

Dyslipidemia is among the comorbidities commonly associated with glaucoma and was seen in approximately 33.6% of our sample. This finding is similar to other global studies but higher than local reports [14,15]. However, several large population studies have shown that dyslipidemia was not significantly related to glaucoma and was negatively associated with the condition. Moreover, statin drugs used to lower cholesterol levels are hypothesized to positively reduce glaucomatous changes [23,24]. On the other hand, several studies have found glaucoma is associated with a higher prevalence of hyperlipidemia [14,25]. Furthermore, there are reports of increased risk of glaucoma in patients taking high doses of statin medications [25].

Visual acuity

Our study reported and categorized the best-corrected visual acuity based on the severity of visual impairment. Each category had an equal distribution between the right and left eye. A majority of the patients in our study had a functional vision that was distributed between patients who had a near-normal vision (19.22%) and normal vision (16.01%). However, 26.87% of the patients had moderate visual impairment. These findings are comparable to a local study that included 124 glaucoma cases which showed that most patients reported mild to no visual impairment (86.3%), which was defined as visual acuity equal to or better than 6/18 [4]. On the other hand, moderate visual impairment was noted in 12.1% of the cases, which was defined as visual acuity worse than 6/18 but better or equal to 6/60 [4].

Limitations

This study was limited by multiple factors, including its small sample size and the retrospective single-center design, limiting the generalizability of results. Moreover, limited documentation in the clinical records is another shortcoming since multiple data were missing. Therefore, population-based studies with larger sample sizes are warranted to delineate better the prevalence and types of glaucoma among the Saudi population.

## Conclusions

Primary open-angle glaucoma was the most common glaucoma subtype affecting half of the patients included in this study. Secondary glaucomas were the second most common, and the majority of cases were found to be neovascular glaucoma. In addition, hypertension and diabetes were the most common reported comorbidities seen in over half the patients. Moreover, a significantly higher proportion of patients had bilateral disease compared to unilateral.
